# Mechanical power and VILI: modeling limits and unknowns

**DOI:** 10.1186/s40635-024-00712-w

**Published:** 2025-01-06

**Authors:** Tommaso Tonetti, John J. Marini

**Affiliations:** 1 Department of Medical and Surgical Sciences (DIMEC), Alma Mater Studiorum University of Bologna, Via Massarenti 9, 40138 Bologna, Italy; 2 Anesthesiology and General Intensive Care Unit, IRCCS Azienda Ospedaliero-Universitaria Di Bologna, Bologna, Italy; 3 Department of Pulmonary and Critical Care Medicine, University of Minnesota, St Paul, Minneapolis, MN USA

**Keywords:** Mechanical ventilation, ARDS, VILI, Mechanical power

Decades of research have brought us closer to identifying the root mechanism of ventilator-induced lung injury (VILI)—tissue damage caused by intolerable tidal parenchymal strain [1]. Total Mechanical power (MP), a measure of inflation energy delivered to the lung parenchyma per minute, has been proposed as a metric that integrates multiple potentially damaging factors (pressure *x* volume *x* frequency) and might aid in developing more ‘lung protective’ ventilatory settings [2]. This makes intuitive sense (energy is needed to effect strain), but while MP clearly serves as an important “big picture” variable, its relationship to VILI causation is far from straightforward and depends on complex interactions within diseased lung tissue.

In this issue of *Intensive Care Medicine Experimental*, Dr. Fabry presents an elegant but rather restrictive mathematical approach to optimizing elastic MP while holding fixed values of minute ventilation and PEEP [3]. Within these constraints and with numerous simplifying assumptions, this exercise examined MP levels under varying levels of tidal volume (VT) and baseline conditions (normal/stiff lung, bigger/smaller artificial airway). The results are then used to underpin firm numerical recommendations for the ideal VT and frequency combination that minimizes elastic MP. Fabry is to be congratulated for his thoughtful and rigorous mathematical approach to solving the intricate puzzle of VILI. However, in addition to the need for a clinical validation of the model, further issues and questions arise.

First, the true risk of VILI in vivo depends on the interplay between the mechanical input from the ventilator settings and intertwined biological/physiological variables, gas exchange and hemodynamics. Moreover, lung stress and strain tend to amplify at the interfaces between regions with different mechanical characteristics, and the number of stress and strain amplifiers (*raisers*) increases in inverse proportion to the size of the ventilatable compartment (*baby lung*) [4]. Of special note, the markedly reduced size of the ‘baby lung’ of ARDS concentrates applied MP and is not considered in Fabry’s model.

Second, the pulmonary vasculature and other key modulators of the VILI response, such as global and local variations of transpulmonary stress, pH and flow amplitude, influence both parenchymal stretch and response. Understandably, these unaddressed factors are not easily incorporated into any simplified mathematical model [4].

Third, Fabry’s approach centers on setting tidal volume based on anatomical dead space (VD_anat_), which is significantly smaller in the “baby” ARDS lung. Relying on standard formulas for VD_anat_ tied to body weight also fails to account for the often-dominant physiological component of total dead space, leaving a critical factor unaddressed [5].

Fourth*,* to estimate the injury risk in response to mechanical forces, the stage of ARDS must be considered, since it influences tissue vulnerability to cyclic stress and strain.

Ultimately, while MP provides a useful framework, it remains an incomplete guide for preventing VILI. The true driver of lung injury is likely the ‘damaging elastic power’ that overstretches vulnerable parenchyma in the ever-lasting pursuit of gas exchange and hemodynamic stability. Emerging technologies like closed-loop ventilation (if properly validated) may help integrate these complexities, adaptively minimizing injurious forces and tailoring therapy to the patient’s evolving condition. To advance our lung-protective strategies, we must move beyond using static variables in our mathematical models to include not only dynamic ones, but also such key factors as lung size, mechanical heterogeneity, hemodynamic impact, and ARDS stage.

## Data Availability

Not applicable.
